# How β-Lactam Antibiotics Enter Bacteria: A Dialogue with the Porins

**DOI:** 10.1371/journal.pone.0005453

**Published:** 2009-05-12

**Authors:** Chloë E. James, Kozhinjampara R. Mahendran, Alexander Molitor, Jean-Michel Bolla, Andrey N. Bessonov, Mathias Winterhalter, Jean-Marie Pagès

**Affiliations:** 1 UMR-MD-1, Transporteurs membranaires, Chimiorésistance et Drug Design, Faculté de Médecine, IFR 88, Université de la Méditerranée, Marseille, France; 2 School of Engineering and Science, Jacobs University Bremen, Bremen, Germany; University of Portsmouth, United Kingdom

## Abstract

**Background:**

Multi-drug resistant (MDR) infections have become a major concern in hospitals worldwide. This study investigates membrane translocation, which is the first step required for drug action on internal bacterial targets. β-lactams, a major antibiotic class, use porins to pass through the outer membrane barrier of Gram-negative bacteria. Clinical reports have linked the MDR phenotype to altered membrane permeability including porin modification and efflux pump expression.

**Methodology/Principal Findings:**

Here influx of β-lactams through the major *Enterobacter aerogenes* porin Omp36 is characterized. Conductance measurements through a single Omp36 trimer reconstituted into a planar lipid bilayer allowed us to count the passage of single β-lactam molecules. Statistical analysis of each transport event yielded the kinetic parameters of antibiotic travel through Omp36 and distinguishable translocation properties of β-lactams were quantified for ertapenem and cefepime. Expression of Omp36 in an otherwise porin-null bacterial strain is shown to confer increases in the killing rate of these antibiotics and in the corresponding bacterial susceptibility.

**Conclusions/Significance:**

We propose the idea of a molecular “passport” that allows rapid transport of substrates through porins. Deciphering antibiotic translocation provides new insights for the design of novel drugs that may be highly effective at passing through the porin constriction zone. Such data may hold the key for the next generation of antibiotics capable of rapid intracellular accumulation to circumvent the further development MDR infections.

## Introduction

One major biological challenge is to understand how the cell controls the exchange of solutes with its environment and to decipher the role of membrane transporters in this process [1], [2]. This aspect of membrane physiology is a key issue in the field of infectious diseases. Antibiotic molecules used in clinical regimens, must penetrate the outer membrane of Gram negative bacteria to reach their target sites and kill the pathogen [3], [4]. Rapid delivery to achieve the required concentrations of antibiotic molecules at their internal targets is now an acute objective due to the threat associated with re-emerging infectious diseases that are resistant to multiple antibiotics. Multi-drug resistant (MDR) bacterial infections have become a worldwide problem, particularly in hospital settings [5]–[7]. Among the most urgent is the opportunistic pathogen, *Enterobacter aerogenes,* responsible for nosocomial infections able to rapidly develop a MDR phenotype within 5 days of antibiotherapy [8]. In order to unlock new therapeutic options/solutions, it is crucial to understand how and how fast antibiotics interact with bacterial cells and the mechanisms that lead to such high levels of resistance. There are 3 major characteristics that an effective antibiotic must exhibit. 1) Rapid and stable accumulation at the target site; 2) Strong target binding; 3) Stability against enzymatic attack [9].

The first step of antibiotic interaction with Gram-negative bacteria is to cross the outer membrane, which forms a protective barrier against hostile environments [3], [4]. The exact mechanism of uptake across this lipid bilayer by hydrophobic compounds is poorly understood. The membrane is punctuated by porins, which are major outer membrane proteins (OMPs) that form water-filled channels allowing diffusion across the membrane. Clinical studies show that the general diffusion porins of many enterobacteriaceal species serve as a major gateway for the passage of β-lactams and fluoroquinolones [3], [4]. Furthermore, alteration of outer membrane permeability, including modification of porin expression has emerged as a major MDR mechanism in *E. aerogenes* and other enterobacterial pathogens [8], [10]–[13]. To improve the translocation efficiency of future antibiotics, it is vital to understand the underlying molecular mechanisms of transport.

The crystal structures of several porins have been determined and the conserved internal loop 3 constitutes a crucial part of the porin channel involved in the influx of antibiotics [4], [14]–[17]. Mutations in this region of Omp36 from *E. aerogenes* and OmpF and OmpC from *E. coli* have been shown to confer altered permeability and susceptibility to various antibiotics [4], [16], [18]–[22]. Analysis of these loop 3 mutations has indicated that certain substitutions induce drastic changes in channel properties due to the presence of bulky or differentially charged residues [20]–[22].

Investigation of antibiotic transport through porin channels can be carried out by insertion of purified porins into planar lipid bilayers. Quantification of the molecular dialogue between antibiotic molecules and porin channels can be achieved *via* analysis of ion current noise in the presence of antibiotics [17], [23]. Measuring the ion current through purified porins reconstituted into planar lipid bilayers provides information about a number of structural and functional properties such as pore size and selectivity [24]. Moreover, the passage of large molecules through the channel interrupt the ion current causing fluctuation or even transient blockages of conductance [17], [23], [25]. Therefore, addition of various antibiotics to the system can cause interaction dependent fluctuations in the ion current and report on the electrophysiological parameters of translocation [17], [25].

In this study transport properties through a major *E. aerogenes* porin, Omp36 (homologous to *E. coli* OmpC and to *Klebsiella pneumoniae* OmpK36) were investigated. Physiological conditions within the patient body favor the expression of Omp36 belonging to the OmpC-family, over OmpF-type porins [10], [16], [26], [27]. This is therefore the more relevant porin type to consider during antibiotherapy [4], [13]. Here the aim was to quantify the influx of representative β-lactams through Omp36. The porin was purified and ion flow through a single trimer reconstituted into a planar lipid bilayer was measured. The presence of antibiotics caused ion current fluctuation in a concentration dependent manner. Analysis of these fluctuations, induced by penetration of the antibiotics into the channel, allowed crucial information to be obtained about the transport mechanism. In addition Omp36 was expressed in the outer membrane of a porin-null *E. coli* mutant (BL21Δ*omp*). Minimum inhibitory concentration assays were used to assess β-lactam susceptibility conferred by Omp36 as the sole porin. Information about the rate of translocation through this porin for delivery to target sites was further provided by measuring the rate of decline of colony forming units following exposure to inhibitory levels of β-lactams.

## Results

### Evidence and quantification of antibiotic translocation through Omp36

**Figure 1:**
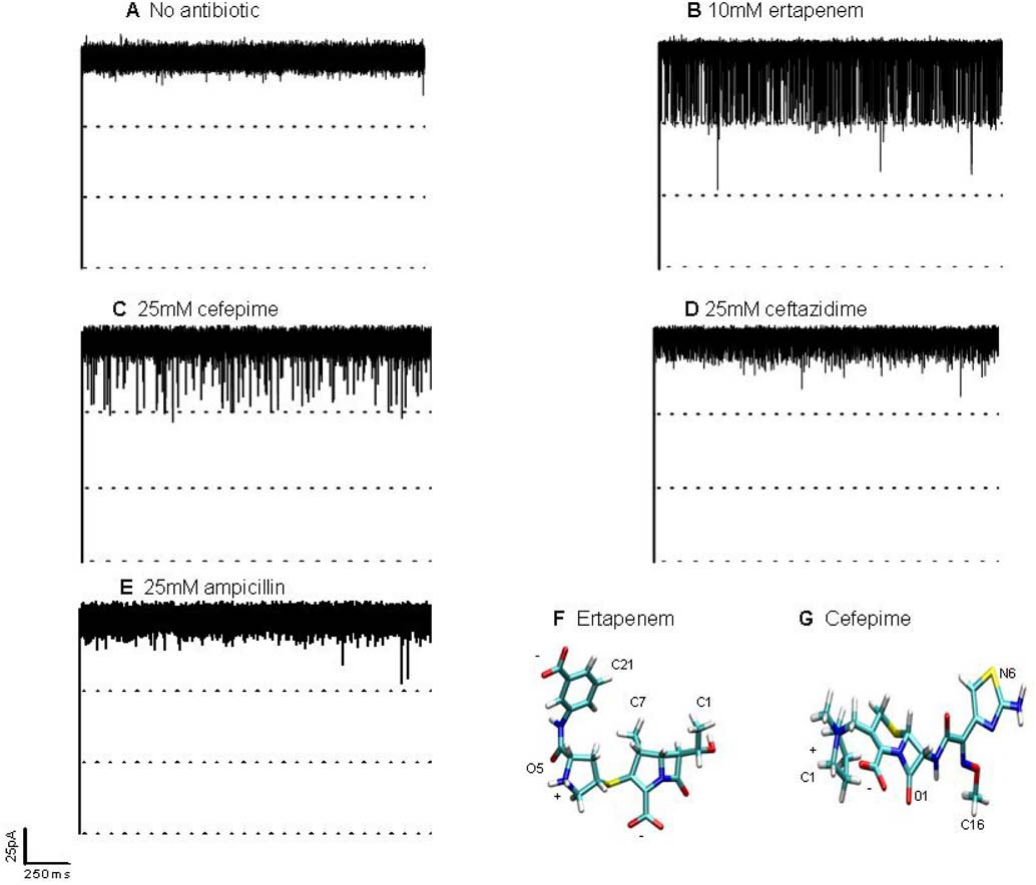
Typical ion current recordings through a single Omp36 trimer reconstituted into planar lipid membranes. a. In the absence of antibiotic, almost no channel closure was visible. b. Addition of 10 mM ertapenem on the *cis* side caused rapid closure of one monomer. c. Addition of 25 mM cefepime on the *cis* side caused significantly less blocking compared to ertapenem. d. Addition of 25 mM ceftazidime on the *cis* side caused no blocking. e. Addition of 25 mM ampicillin on the *cis* side caused no blocking. Membrane bathing solution was 1 M KCl (pH 6) and the applied voltage was 50 mV. Chemical structure of antibiotics. f. Ertapenem g. Cefepime. The antibiotics are displayed in ”balls and sticks” and colored by atom type (oxygens in red, nitrogens in blue, carbons in cyan, sulfur in green, hydrogens in white).

The *omp36* gene was cloned and expressed in the porin-null *E. coli* strain BL21Δ*omp* [28] (see **Text S1**, **Table S1** and **Fig. S1**). Omp36 was purified using ion-exchange chromatography and a single trimeric porin was reconstituted into artificial lipid membranes [17], [23]. Application of a transmembrane voltage established an ion current through the channel and, in the absence of antibiotics, no visible current blockage was detected up to a voltage of <150–200 mV **(** **Fig. 1a** **)**. Addition of antibiotics to this system caused fluctuations in the ion current reflecting the possible channel-drug interactions. Ertapenem which is a negatively charged carbapenem [9] caused spontaneous blockage of the ionic currents **(** **Fig. 1b** **)**. The presence of antibiotic caused rapid blockages of the monomers. These ion current fluctuation increased with increasing concentration (see **Text S1** and **Fig. S2**). Furthermore, analysis at higher time resolution clearly indicated complete monomer channel blockages. On average 0.5 mM ertapenem caused single monomer blockages and at an increased concentration of 15 mM two monomers were blocked. Interactions with cefepime, a zwitterionic cephalosporin [9], were also detected, but the blockage events were shorter and less frequent than those caused by ertapenem **(** **Fig. 1c** **)**. In contrast ceftazidime **(** **Fig. 1d** **)** and ampicillin **(** **Fig. 1e** **)** caused no significant blockage of the ionic current indicating negligible interaction with the channel. Similar characterization of OmpC, for which a high-resolution structure has recently been resolved [15], showed the same pattern of interaction with ertapenem, cefepime, ceftazidime and ampicillin (data not shown).

**Figure 2:**
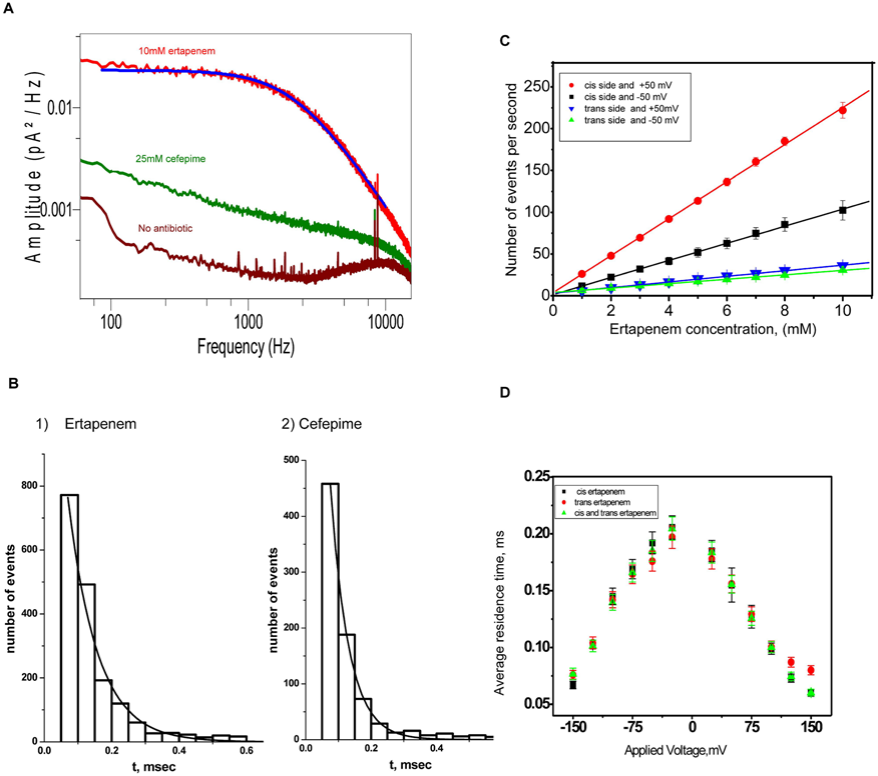
Kinetic analysis of antibiotic transport through Omp36. a. Power spectral densities of the excess noise in the ion current through a single trimeric Omp36 channel in the presence two different antibiotics ertapenem and cefepime added to the *cis* side of the lipid membrane. Smooth solid line through the spectra is Lorentzian fit with τ = 120 µs for ertapenem. b. Time histogram of Omp36 channel blockage in presence of 10 mM ertapenem (1) or 25 mM cefepime (2) added to the *cis* side of the lipid membrane. Solid line is the single exponential fit with characteristic time τ = 128 µs(1) and 105 µs(2). c. The number of ertapenem blocking events per second was linear to ertapenem concentration and depended on applied voltage and side of antibiotic addition (*cis* or *trans* side). d. Ertapenem residence time did not depend on the direction of the drug addition (*cis*, *trans* or both sides) and it depended on the applied voltage. Average residence time decreased with increased applied voltage.

The penetration of antibiotics into the channel can also be measured by analysing the power density spectra of the ion current. In **Fig. 2a**, a typical power density spectra of the ion current fluctuations is shown. The figure shows clearly the effect of different antibiotics: the presence of 10 mM ertapenem increased the ion current noise 15 fold compared to background levels. In contrast a much higher concentration of cefepime (25 mM) caused only a doubling of the noise level. In the case of ampicillin and ceftazidime (see **Text S1** and **Fig. S3**) no excess noise was visible (see Material and Methods for details). As previously shown the excess noise was caused by perturbing the ion current inside the channel in the presence of interacting antibiotics [17], [23]. Channel blocking by the antibiotic molecules was also quantified by using a statistical analysis of the channel in its ‘un-occupied’ and ‘occupied’ (or blocked) states. The average residence time (τ) of each antibiotic in the Omp36 channel was obtained by single exponential fitting of blockage time histograms with the distribution of dwell time in the blocked state (single channel analysis). The τ was 0.14±0.02 ms for ertapenem and 0.10±0.02 ms for cefepime at 50 mV (**Fig. 2b**) (see Material and Methods for details).

The strength of the ertapenem interactions allowed further quantification of the “molecular dialogue” between this antibiotic and the Omp36 channel. **Fig. 2c** shows the number blocking events, which increase with increasing antibiotic concentration. Following *cis* side addition of ertapenem more intense blocking was observed compared to *trans* side addition. This indicated an asymmetry in accessibility of the binding site and a lower energy barrier from the *cis* side. As ertapenem is negatively charged, *cis* side addition of the antibiotic along with the application of positive voltages should favor translocation through the channel. Clearly, we observed an enhanced rate of translocation when the antibiotic was travelling from the *cis* to the *trans* side. However, blockage events following *trans* side addition of ertapenem were less frequent and voltage independent. This indicated a higher energy barrier on the *trans* side and the electric field did not influence the rate of translocation. In **Fig. 2d**, the average residence time of antibiotic molecules in the channel measured at different voltages is shown. It is important to note that the residence time was independent of the concentration and of which side of the membrane the drug was added. This indicated the presence of a single affinity site in the channel according to the model described by Schwarz *et al.*, 2003 [25].

We simplified the mathematical analysis by assuming the presence of one affinity site within the channel, accessible from both sides of the lipid membrane. The most important step that determines antibiotic translocation is the entrance and exit rates. The kinetic rate for channel entrance and exit allows estimation of the net flux of antibiotics. For example when 1 mM ertapenem was added to the *cis* side of the lipid membrane, the association rate constant (k_on_) was 9100±1000 (Ms)^−1^ and the binding constant (K) was 1.50±0.05 M^−1^ at an applied voltage of 50 mV. As previously shown the flux of antibiotic molecules per second is given by [25], [29], [30] (see Material and Methods for details)
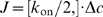
This analysis concluded that, using a 1 mM ertapenem concentration gradient across the channel, about 5 molecules were able to translocate each Omp36 monomer per second. Blocking events in the presence of cefepime were weak compared to ertapenem. When 1 mM cefepime was added to the *cis* side of the lipid membrane, the association rate constant (k_on_) was 1000±100 (Ms)^−1^ and the binding constant (K) was 0.2±0.02 M^−1^ at an applied voltage of 50 mV. The number of molecules translocated was approximately 0.5 molecules per second per monomer. Inspection of the above equation showed that translocation was proportional to the on-rate [25], [29], [30]. The strength of an antibiotic interaction with the affinity site of a channel greatly influences the efficacy of its translocation [31], [32]. Our data shows that ertapenem interacts more strongly than cefepime with the Omp36 channel (**Fig 1** **,** **2**) and translocates the channel more rapidly.

### Rate of β-lactam Action on *E. coli* Expressing Omp36 as the Sole Porin

**Figure 3:**
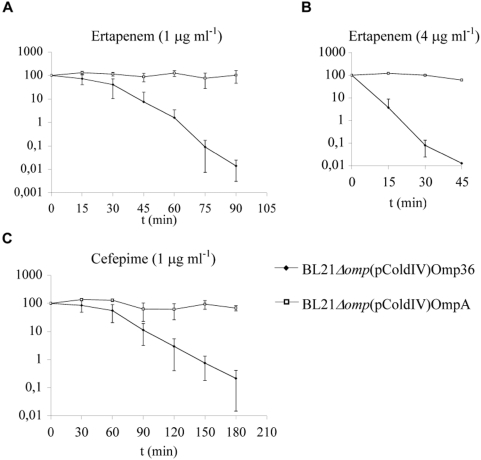
Influence of Omp36 Expression on the Rate of β-lactam Antibiotic Activity. Percentage decrease in cfu ml^−1^ of BL21*Δomp* cultures expressing either Omp36 or OmpA following exposure to inhibitory concentrations of: a ertapenem (1 µg ml^−1^), b ertapenem (4 µg ml^−1^), c cefepime (1 µg ml^−1^). Experiments were repeated three times and error bars were indicated.

The ability of β-lactams to traverse the outer membrane *via* Omp36 channels was initially determined here using minimum inhibitory concentration (MIC) assays. Omp36 (or OmpA as a negative control – see **Text S1**) was expressed, on an IPTG inducible plasmid, as the sole porin in an otherwise porin-null *E. coli* strain (BL21Δ*omp*). Expression of Omp36 in the outer membrane (see **Text S1** and **Fig. S1**) resulted in an 8 fold increase in sensitivity to ertapenem with an MIC of 0.5 µg ml^−1^ in IPTG-induced cultures compared to 4 µg ml^−1^ in non-induced cultures and those harboring vector only (see **Text S1** and **Table S1**). These data confirmed the involvement of Omp36 in β-lactam susceptibility (see **Text S1**). We further compared the efficacy of ertapenem and cefepime action by exposing bacterial cultures to inhibitory concentrations of each β-lactam and observing the percentage decreases in cell number (colony forming units, cfu ml^−1^) over time (**Fig. 3**). In the presence of either ertapenem or cefepime, BL21Δ*omp* cultures expressing Omp36 as the sole porin were depleted at a dramatically increased rate compared to those expressing OmpA (**Fig. 3**) and, to a lesser extent, vector only (data not shown). The action of ertapenem was observed to be considerably faster than cefepime with a 90% decrease in cfu ml^−1^ of Omp36 expressing cultures within 45 minutes and 90 minutes respectively and a 99% decrease within 60 minutes and 150 minutes. Care must be taken when interpreting this data. The rapid action of ertapenem could be attributed to high target affinity or stability against β-lactamase degradation [33], [34] (see Supplementary Data Section). However, with the use of stringent controls imposed here, these results corroborate both MIC and electrophysiological data, suggesting that efficient interactions of ertapenem with an affinity site in the Omp36 channel confer faster influx across the outer membrane *via* this porin, contributing to the faster rate of action.

## Discussion

This study deciphers a role for the enterobacterial porin, Omp36 in antibiotic transport. Recent clinical studies of *K. pneumoniae* infection observed that exposure to ertapenem promoted drug resistance *via* the loss of OmpK36 [36]–[38]. Furthermore, many recently evolved metallo-carbapenemases participating in the enzymatic barrier require decreased porin expression to effectively confer high-level carbapenem resistance [33]. Increasing clinical studies report the down-regulation of porin expression, or a shift favoring the expression of smaller or more restrictive channels, as a response to antibiotherapy [4], [13]. This results in reduced membrane permeability that severely limits intracellular drug accumulation, allowing the evolution and/or the acquisition of other resistance mechanisms including target mutations, enzymatic production, etc [13]. Such reports highlight the importance of: 1) efficient influx through porins for β-lactams to reach their target sites, and 2) a detailed understanding of this dynamic and interactive process.

The pathway of the antibiotic molecule through the channel is of crucial importance for the intracellular accumulation of antibacterial drugs. It has become clear that the transport of β-lactams or fluoroquinolones through OmpF-type porins is not by passive diffusion through an inert tube, but involves specific interactions with porin channels [17], [19], [23]. Due to the detailed knowledge of its crystal structure most studies of antibiotic-porin interactions so far have focused on OmpF from *E. coli* [14], [39], which is a major porin type expressed *in vitro* along with homologs Omp35 and OmpK35 in *Enterobacter* and *Klebsiella* spp. However, *in vivo* temperature and salt concentrations, favor the expression of OmpC-type porins including *Klebsiella pneumoniae* OmpK36 and *E. aerogenes* Omp36 [26], [27] investigated here. Consequently, these are the dominant porins in the patient body [3], [4], [13] and represent the key strategic pathways for β-lactams and fluoroquinolones to penetrate the bacterial cell during patient therapy. Our study combines high resolution ion conductance measurements with biological susceptibility assays to explore β-lactam translocation properties through Omp36, a major porin of the MDR pathogen, *E. aerogenes*. Using two representative β-lactam molecules, we demonstrate that interaction with the channel correlates with facilitated translocation through the porin and thus enhances the transport efficiency. We hypothesize that there is a strong interaction, involving hydrophobic and hydrogen bonds, between ertapenem and specific aminoacid residues which constitute the affinity site within Omp36. Ertapenem has a net negative charge and two carboxylic groups are able to form hydrogen bonds with the basic residues of the channel. In the case of cefepime (a zwitterionic compound) we measured a lower channel affinity. This is in agreement with previous molecular modeling of cefepime in the constriction zone of OmpF [19] which is the Omp35 homologue in *E. coli* [4]. For optimal permeation, a balance is required between affinity and repulsion interactions at key sites in the constriction zone. Our MIC data agree with the electrophysiological results, showing stronger activity of ertapenem than cefepime in bacterial cells expressing Omp36 as the sole functional porin. In addition we have demonstrated the rate of ertapenem antibiotic action on these cells to be strongly faster than that of cefepime and that this is partly due to more rapid transport through the porin.

A number of chemical and physical properties of antibiotic molecules, such as size, hydrophobicity, stoichiometry and charge, have been shown to influence their rate of permeation through porin channels. For example, zwitterionic compounds have been shown to penetrate proteoliposomes very rapidly [40] and have induced increased ion flux perturbations through OmpF in lipid bilayer models compared to other charged compounds. In addition, large molecules, with bulky side-chains, such as azlocillin and piperacillin have shown low permeation rates [17].

Efficient translocation through porins requires favorable channel properties in addition to a streamlined antibiotic molecule. As β-lactam molecules are similar in size to the channel diameter, their passage is not a simple diffusion but rather a gliding process along the pore wall. Within the constriction zone of porin channels, strategically located residues create a strong electrostatic field [15], [17], [36]. Key exposed residues particularly in the internal loop 3 have been identified that transiently interact with translocating molecules to strongly influence the rate of permeation [17] and the antibiotic efficacy [19]. Site-directed mutagenesis at such sites in *E. coli* OmpF and OmpC has been shown to alter susceptibility to certain antibacterial molecules [21], [22], [41], [42]. OmpC and Omp36 porins harboring loop 3 mutations have been detected in a small number of resistant clinical isolates of *E. coli* and *E. aerogenes* and may represent an emerging bacterial drug resistance strategy in order to restrict antibiotic influx [4], [13]. Several biophysical investigations report the interaction between ampicillin and OmpF during drug diffusion in agreement with microbiological evidence [17], [19], [24]. In contrast, we have shown that ampicillin interaction with Omp36 and OmpC is negligible. Nikaido and Rosenberg [43] showed much restricted penetration of antibiotic molecules with bulky side-chains and negative charges through OmpC than through the wider OmpF channel. The recently resolved OmpC crystal structure suggests that electrostatic pore potential and specific atomic details inside the channel are the key parameters distinguishing OmpC and OmpF rather than size [15]. This reduced permeability through OmpC-type porins could explain the shift from OmpF-type to OmpC-type expression observed in clinical isolates during antibiotherapy as a strategy to limit antibiotic influx [4], [13]. Ertapenem and cefepime both possess some of the star qualities required for rapid translocation. They are small and compact, and interact with the channel significantly. The recent description of the OmpC 3D structure [15], presents the opportunity to decipher some of the detailed molecular criteria involved in antibiotic diffusion through this porin group. Future experiments should explore mutagenesis of key sites within the Omp36 L3 loop to decipher exactly which residues are interacting with each drug, and therefore, which aspects of the antibiotic molecular structure drives rapid transport.

Our data suggest that for optimal permeation, a balance is required between affinity and repulsion interactions at key sites in the constriction zone. Consequently, the strength of interaction has a major influence on rates of antibiotic penetration, *ie* intracellular accumulation, and thus antibiotic efficiency [13].

A combination of efficient intracellular accumulation, stability against β-lactamases and target affinity is exhibited by ertapenem for effective antibiotic activity in bacteria. Crossing the outer membrane is the first step in the β-lactam journey to its periplasmic target site ensuring sufficient intracellular concentrations for bacteriocidal activity. We report here that certain molecular characteristics such as compact structure and a particular pattern of ionic charges yet to be deciphered may constitute a ‘passport’ for rapid travel through the porin demonstrating that drug passive diffusion is in fact an interactive process. Our approach may contribute to the rational design of new antibiotic candidates against MDR pathogens and serve to optimize influx by screening translocation rates of new compounds, to determine whether they hold a valid passport for the most efficient delivery to target sites.

## Materials and Methods

### Bacterial Strains and Culture Media

Cloning was performed using *E. coli* JM109. Protein expression for purification and MIC experiments was performed in porin-null *E. coli* BL21(DE3)omp8 (Δ*lamB, ompF::Tn5,* Δ*ompA,* Δ*ompC* ) referred to in the text as BL21Δ*omp* [28]. Bacteria were grown in Luria bertani (LB) broth (Difco) except during MIC experiments, in which Muller Hinton (MH) broth (Difco) was used. Transformants were selected on Luria Bertani agar (Difco) containing relevant antibiotics (kanamycin (50 µg ml^−1^) and or ampicillin (100 µg ml^−1^) (Sigma)).

### Cloning and Outer Membrane Expression of *omp36* and *ompA*

The *omp36* (1137 bp) and *ompA* (1085 bp) genes were amplified, including their signal peptide sequences, from *E. aerogenes* ATCC strain 13048 using PCR, and restriction sites were added (underlined in the primer sequence) to each end using primers *5′omp36Bam*HI (5′-GTTAGCGGATCCATGAAAGTTAAAGTACTGTCCCTC 3′) and 3′*omp36Hind*III (5′-GCGTTAGCAAGCTTCAGCGTGCTTAGAACTGGTA-3′) and *5′ompABam*HI (5′-GTTAGCGGATCCATGAAAAAGACAGCTATCGC-3′) and 3′*ompAHind*III (5′-GCGTTAGCAAGCTTGGAAACTTAAGCCTGCG-3′) respectively. PrimeSTAR™HS DNA polymerase (Takara) was used to amplify products by PCR according to the manufacturers instructions (cycling conditions; melting at 98°C, 10 s; annealing at 58°C, 10 s, extension at 72°C, 60 s). Purified PCR products were digested using *Bam*HI and *Hind*III (New England Biolabs) and cloned into the expression vector pColdIV (4359 bp) (Takara), using T4 Ligase (NEB) to create pColdIV*omp36* and pColdIV*ompA*. Plasmid constructs were confirmed by sequencing (GenomeExpress), using the primer pair pColdF (5′-ACGCCATATCGCCGAAAGG-3′) and pColdR (5′-GGCAGGGATCTTAGATTCTG-3′) [44] then transformed into BL21Δ*omp*. Transformants were grown to early-exponential phase (OD_600_ 0.4) in LB at 37°C before chilling to 15°C and adding 1 mM IPTG (Eurogentec) for 18 hours. Expression was confirmed by SDS PAGE and immunodetection.

### Minimum Inhibitory Concentration Assays

BL21Δ*omp* cultures harboring pColdIV, pColdIV*omp36* or pColdIV*ompA*, were grown to OD_600_ 0.4 in LB containing appropriate antibiotics. Cultures were split into 2 flasks, 1 was induced with IPTG (1 mM) for 1 h and the other was not. Bacteria were then subcultured into MH broth with or without IPTG (0.5 mM) and β-lactamase quenchers tazobactam, clavulanic acid and cloxacillin (4 µg ml^−1^ each) at OD_600_ 0.001 containing no antibiotics. 2-fold dilution series of each antibiotic studied were prepared and added to 1 ml aliquots of bacterial suspensions in MH. Assays were incubated for 18–24 h, 37°C. Each assay was repeated independently 3 times and results were classified according to the Antibiogram Committee of the French Society for Microbiology (http://www.sfm.asso.fr).

### Rate of Antibiotic Action Assays

BL21Δ*omp E. coli* cultures harboring either pColdIV*omp36* or pColdIV*ompA*, were prepared as for MIC assays. In trials performed using the MIC for cultures producing OmpA (4 µg ml^−1^), Omp36 expressing cultures were depleted to un-detectable levels within 20 min (see **Text S1** and **Fig. S1**). In order to accurately quantify the rate of action over a number of time points, all induced and diluted cell suspensions (OD_600_ 0.01) were exposed to 2× the MIC for cultures producing Omp36 (1 µg ml^−1^). At 15–30 min time intervals, 10-fold dilution series of exposed cultures were prepared with LB and spread onto LB agar containing appropriate antibiotics. Plates were incubated overnight at 37°C for 18 h and colonies were counted. Colony forming units (cfu/ml) were calculated for each time point and plotted as the percentage decrease in cfu/ml compared to t = 0. All experiments were repeated independently at least 4 times.

### Outer Membrane Extraction

The method for extracting outer membranes (OM) was modified from Bolla [45]. Briefly, induced cultures (1 L) were harvested by centrifugation (10,000×g, 20 min, 4°C). Bacterial cells were disrupted in 50 mM sodium phosphate buffer, (NaPi) pH 7.4 by sonication using the Branson Sonifer 450 (7×2 min, output level 5) on ice and total membranes collected by ultracentrifugation (100,000×g, 1 h, 4°C). Inner membrane proteins were solubilized by agitation with sodium lauryl sarcosinate, 0.15% w/v (sigma) in NaPi (50 mM, pH 7.4, room temperature, 30 min). OM proteins were harvested by ultracentrifugation (100,000×g, 1 h, 4°C). OM expression of Omp36 was assessed using SDS PAGE and immunodetection.

### SDS PAGE and Western Blotting

Bacterial protein extracts were analyzed on SDS-PAGE gels containing 10% acrylamide. Samples were denatured in Laemmli loading dye containing 2% SDS and heated 3× to 95°C [11]. Protein size was estimated by comparison with pre-stained low-range molecular weight marker (BioRad). Proteins were stained using Coomassie Brilliant Blue R-250.

For immunodetection, proteins were electrotransfered onto nitrocellulose membranes (Schleicher & Schlull, Keene, NH, USA) in transfer buffer (20 mM Tris, 150 mM glycine, 20% isopropanol, 0.05% SDS). Membranes were blocked using 4% milk in Tris-buffered sodium (TBS: 50 mM Tris-HCl, 150 mM NaCl, pH 8). Polyclonal antibodies were used for detection, anti-F4 antibody directed against a small peptide of the conserved internal loop 3 for porin and anti-OmpA antibody directed against OmpA of *E. coli* for OmpA [11], [18]. Detection of antigen-antibody complexes was performed with alkaline phosphatase-conjugated AffinitiPure goat anti-rabbit IgG antibodies (Jackson ImmunoResearch, West Grove PA, USA) using BCIP and NBT (Sigma) according to the manufacturers instructions.

### Purification of Omp36

Purification methods were developed from Bolla [45] and Garavito and Rosenbusch [46]. OM extracts were washed with 0.5% octyl-POE (Bachem AG, Bubendorf, Switzerland) in NaPi (50 mM, pH 7.4). Selective extraction of Omp36 was performed by solubilization from OM preparations using 1% octyl-POE+NaCl (1 M) at 37°C, 1 h with shaking. Unsolubilized proteins were removed by ultracentrifugation (100,000×g, 1 h at 4°C). Extraction from the pellet was repeated twice using the same conditions. Supernatants were pooled and concentrated using YM-30 centricon filters and NaCl was removed using Hi-Trap de-salting columns (GE Healthcare). Omp36 was purified from solubilized protein extracts using a Resource Q ion exchange column (Amersham Biosciences). The column was equilibrated with NaPi, pH 7.4 containing 1.2% POE and 10 mM NaCl. Extracts were loaded at a flow rate of 2 ml min^−1^, monitoring conductivity and OD at 280 nm at all times using Akta Explorer 10 apparatus. Omp36 was eluted from the column using a linear gradient (12 CV) from 10 mM to 1 M NaCl. Fractions containing Omp36 were verified by SDS-PAGE and immonoblotting.

### Single channel measurements and antibiotic interaction

Virtually solvent-free lipid bilayer membranes were formed as previously described by Montal and Mueller [47]. To form planar lipid bilayers with the monolayer opposition technique, we used 1,2-Diphytanoyl-sn-Glycero-3-Phosphatidylcholine (Avantipolar lipids). Two symmetrical compartments of a Teflon chamber each with a solution volume of 0.25 ml of KCl (1 M, pH 6) were separated by a 25 µm thick Teflon film (Goodfellow, Cambridge,UK) containing a round aperture of 60–80 µm diameter. The aperture was pretreated with 1% hexadecane in pentane. Ag/AgCl electrodes were used to detect ion currents (World Precision Instruments, Sarasota FL, USA). The *cis* electrode was grounded while the *trans* electrode was connected to the head stage of an Axopatch 200B amplifier (Axon Instruments, Foster City, CA). The applied membrane voltage refers to the difference between the *cis* and *trans* side potentials. The membrane capacitance was 50–100 picofarads. Single channel insertion was achieved by adding 1–2 µl of Omp36 extract (18 ng ml^−1^) containing 0.6% Octyl POE to the chamber. Single channel insertion was facilitated by applying a membrane voltage of 200 mV and mixing the contents of the chamber. Measurements were performed with an Axopatch 200B amplifier in the voltage clamp mode. Under the applied voltage, protein insertion was easily detected by current increase. The porin was always added to the *cis*-side of the chamber. It is interesting to note that single porin insertion was always asymmetric in contrast to multi-channel recording leading to a more equally distributed orientation. Channel conductance is slightly higher at positive voltage compared to negative voltage in all experiments, which can be used as the test for the direction of channel insertion. Signal was filtered using a low-pass Bessel filter at 10 kHz and recorded to PC at 50 kHz sampling frequency. Data analysis was performed using Clampfit software (Axon Instruments, Inc.). All experiments were carried out at room temperature.

Ion current fluctuations in the presence of various antibiotics were measured at an applied transmembrane voltage. Concentrated aliquots of antibiotic solutions were added to the lipid chamber, mixed very well, and incubated for 10 minutes for complete diffusion in the chamber prior to recording. Antibiotic stock solutions were prepared in 1 M KCl buffered by MES. The pH of the solution was measured and adjusted after the preparation of the stock solution and continuously measured at different concentrations in the course of the experiment and after completing the experiment. Blockage events occurred following addition of antibiotics ertapenem and cefepime to either the *cis* or *trans* side of the artificial membranes. These blockages reveal the current state of the “binding” site and allow analysis of the occupation on a single molecular level. The first step is to analyse the statistic of the time histogram. If the interaction of the antibiotic with the channel can be described by a simple two-state Markovian (no hysteresis) a single exponential decay is observed. The average residence time of antibiotic was calculated using single exponential fitting of blockage time histograms (**Fig. 2b**). At low concentration, [c] ≪k_off_/k_on_, the characteristic time was close to the average residence time of the drug (τ) thus allowing us to use the following equations: τ≈k_off_^−1^, and k_on_ = v/(3[c]) where v is the number of binding events and [c] was the antibiotic concentration. A similar approach was employed for the estimation of ampicillin and moxifloxacin translocation rates through the *E. coli* OmpF channel [24], [31].

In the case where single blockage events are less pronounced, the power density spectra is more suited to analyse interactions [29]. Electronically it is rather favorable to average over typical occurring frequencies and the above exponential decay will lead to a Lorentzian in the power density spectra. The experimental parameter is the corner frequency at which a Lorentzian decayed to half of its original value ω_c_ = k_on_ [c]+k_off_.

The spectrum of ion current fluctuations was fitted to the Lorentzian model: *S* (*f*) = *S* (0)/(1+(*f*/*f*_c_)^2^), where *S* (0) was the low-frequency spectral density and *f*_c_ was the corner frequency, giving the relaxation time constant defined as τ* = *1/2π*f*_c_. It is interesting to note that the concentration dependent corner frequency obtained from a Lorentzian fit of the power spectrum yielded the same results (data not shown). The corner frequency increased in a concentration dependent manner allowing determination of the on and off rates of ertapenem into the affinity site of the Omp36 channel. In contrast only little increase was visible for cefepime and none for ampicilin and ceftazidime.

## Supporting Information

Text S1 Supplementary text S1
(0.04 MB DOC)

Table S1 (0.02 MB PDF)

Figure S1 (0.45 MB EPS)

Figure S2 (0.11 MB EPS)

Figure S3 (0.11 MB EPS)
