# Supplementary material for: How β-Lactam Antibiotics Enter Bacteria: A Dialogue with the Porins
Source: PLoS One. 2009 May 12;4(5):e5453. doi: 10.1371/journal.pone.0005453 (PMC2677626; doi:10.1371/journal.pone.0005453)
Supplement: Text S1 — Supplementary text S1 (0.04 MB DOC) [file pone.0005453.s001.doc]

TEXT S1

(supplementary data section)

How ß-lactam Antibiotics Enter Bacteria : A Dialogue with the Porins

Chloë E. James^†1^, Kozhinjampara R. Mahendran^†2^, Alexander Molitor^1^ Jean-Michel Bolla^1^, Andrey N. Bessonov^2^, Mathias Winterhalter^2^, Jean-Marie Pagès^1^*

^1^ UMR-MD-1, Transporteurs membranaires, Chimiorésistance et Drug Design, Faculté de Médecine, IFR 88, Université de la Méditerranée, Marseille, France

^2^ School of Engineering and Science, Jacobs University Bremen, Campus Ring 1, Bremen, Germany

**Omp36 plays a role in antibiotic susceptibility**

The ability of β-lactams to traverse the outer membrane *via* Omp36 was classically determined using minimum inhibitory concentration (MIC) assays [http://www.clsi.org/]. The porin-null E. coli strain BL21Δomp was transformed with the IPTG inducible expression vector, pColdIV, into which omp36 had been cloned. BL21Δomp expressing Omp36 in the outer membrane (**Fig. S1**) exhibited an 8 fold increase in sensitivity to ertapenem with an MIC of 0.5 μg ml^-1^ in IPTG-induced cultures compared to 4 μg ml^-1^ in non-induced cultures and those harboring vector only (**Table S1**).

Cefepime was found to be sensitive to degradation by a β-lactamase encoded by the amp selective marker of the pColdIV vector. Therefore, a more sophisticated approach was required to determine the true activity of this antibiotic. In the presence of enzyme inhibitors to quench the β-lactamase action, cells expressing Omp36 showed a 4 fold increase in sensitivity to cefepime from 2 μg ml^-1^ to 0.5 μg ml^-1^. The β-lactamase inhibitors did not affect the MIC of ertapenem, which was resistant to the amp-encoded enzyme [33, 34].

The complete lack of major outer membrane proteins (e.g. OmpC, OmpF, OmpA) in E. coli strains such as BL21Δomp may alter outer membrane stability and could result in a “leaky” phenotype and loss of fitness. We therefore decided to include an internal control in which a foreign OMP was produced, that may contribute to membrane stabilization. Expression of the major enterobacterial protein, OmpA, which is known to participate in outer membrane structure was selected [3,35]. The E. aerogenes ompA gene was cloned and expressed in BL21Δomp in exactly the same manner as omp36. Immunoblots confirmed that Omp36 and OmpA were correctly processed to the outer membrane at similar levels when their respective expression was induced with IPTG (**Fig. S1**). Outer membrane expression of OmpA showed no effect on the level of antibiotic susceptibility (**Table S1**) demonstrating that OmpA was not involved in antibiotic membrane translocation and that induced expression of a plasmid-encoded OMP did not alter the membrane permeability. This confirmed that the observed increase in antibiotic sensitivity in cells expressing Omp36, was due to the passage of antibiotics through the porin.

###### Ertapenem dose effect on pore conductance

In order to analyse the ß-lactam dose effect on channel activity, we measured ion current fluctuations over a wide concentrations range – up to 15 mM for ertapenem and to 25 mM for other antibiotics (higher concentrations were limited by solubility thresholds). Various ertapenem amounts were used (0.5 mM, 2 mM, 5 mM and 7 mM) in addition to 10 mM (presented in **Fig. 1)**. **Fig. S2** clearly illustrates that ertapenem blocks the passage of the ionic currents in a concentration dependent manner.

**Noise analysis in the presence of ampicillin and ceftazidime**

**Fig. S3** demonstrates that no fluctuations in the ionic current through Omp36 were observed in the presence of ampicillin and ceftazidime under the same conditions used for ertapenem and cefepime titrations (**Fig. 2a)**.

**Figure legends**

**Figure S1**

**SDS-PAGE and Immunoblot Analysis of Omp36 and OmpA Expression.**

**a**. Coomassie stained SDS-PAGE showing expression of OMPs in outer membrane extracts of *E. coli* BL21*Δomp* harboring pColdIV*omp36* (lane 1); pColdIV*ompA* (lane 2) or pColdIV only (lane 3). **b**. Immunoblot showing Omp36 and OmpA in outer membrane extracts of *E. coli* BL21*Δomp* harboring pColdIV*omp36* (lane 1); pColdIV*ompA* (lane 2). **c.** Immunoblot using **i)** anti-F4 or **ii)** anti-OmpA antibodies. Lanes 1-3 total cell extracts from BL21*Δomp* cultures harboring **i)** pColdIV*omp36* or **ii)** pColdIV*ompA* exposed to various IPTG induction conditions (-- non-induced cultures; -+ 1h IPTG induction; ++ Continuous exposure to IPTG, 18 h); Lane 4, cells containing pColdIV vector only under continuous IPTG exposure.

**Figure S2**

**Blockage of the ionic currents in a concentration dependent manner**

Measurements of ion currents through Omp36 were carried out at various concentrations range for ertapenem (0.5mM, 2 mM, 5 mM and 7 mM) in addition to 10 mM presented in Fig.1 under the same experimental conditions.

## Figure S3

**Noise analysis with other ß-lactam antibiotics**

Power spectral densities of noise in the ion current through a single trimeric Omp36 channel in the presence of ceftazidime and ampicillin added to the *cis* side of the lipid membrane.
