# Supplementary material for: How β-Lactam Antibiotics Enter Bacteria: A Dialogue with the Porins
Source: PLoS One. 2009 May 12;4(5):e5453. doi: 10.1371/journal.pone.0005453 (PMC2677626; doi:10.1371/journal.pone.0005453)
Supplement: Table S1 — (0.02 MB PDF) [file pone.0005453.s002.pdf]

Table S1

MIC values (μg ml $^{-1}$) for *E. coli* strain BL21*Δomp* harboring the pColdIV vector + /- *omp* gene in the presence of IPTG (0.5 mM)

Vector	IPTG	ERT	FEP + CTC	FEP - CTC
pColdIV only	+/-	4	2-4	4
pColdIV*omp 36*	-	4	2	4
pColdIV*omp 36*	+	0.5	0.5	4
pColdIV*ompA*	-	4	2	4
pColdIV*ompA*	+	4	4	8

Experiments were repeated 3-5 times

IPTG (Isopropyl-beta-thio-galactoside); ERT (ertapenem); FEP (cefepime); CTC (clavulanic acid, tazobactam and cloxacillin).
